# Proposed Role of Circadian Clock Genes in Pathogenesis of HCC: Molecular Subtyping and Characterization

**DOI:** 10.3390/biomedicines14030645

**Published:** 2026-03-12

**Authors:** Zhikui Lu, Yi Zhou, Jian Luo, Zhicheng Liu, Zhenyu Xiao

**Affiliations:** 1 Division of Hepato-Pancreato-Biliary Surgery, Tongji Hospital, Tongji Medical College, Huazhong University of Science and Technology, Wuhan 430030, China; 2 Department of Geriatrics, Tongji Hospital, Tongji Medical College, Huazhong University of Science and Technology, Wuhan 430030, China

**Keywords:** hepatocellular carcinoma, circadian clock genes, molecular subtyping, immune microenvironment, metabolic reprogramming, therapeutic sensitivity

## Abstract

**Background:** Hepatocellular carcinoma (HCC) stands as a prevalent global health issue with increasing incidence and mortality rates. Hepatocellular carcinoma (HCC) exhibits profound molecular and clinical heterogeneity, which limits the effectiveness of current therapeutic strategies. Circadian rhythm disruption has been implicated in metabolic reprogramming, proliferation, and immune modulation in cancer, but its role in shaping HCC heterogeneity remains poorly defined. **Methods:** Four public HCC transcriptomic cohorts (TCGA-LIHC, CHCC, LIRI, LICA) were integrated using RMA normalization and ComBat for batch correction. Consensus clustering based on 31 core circadian clock genes (CCGs) identified robust molecular subtypes. Multi-omics characterization—including genomic alterations, pathway activity (GSEA/GSVA), immune microenvironment profiling (CIBERSORT, EPIC, MCP-counter, xCell), and drug-sensitivity prediction (pRRophetic/oncoPredict)—was performed to delineate subtype-specific biological properties. A nine-gene CCG-based RiskScore model was constructed using LASSO Cox regression to internally validate subtype robustness and intra-subtype risk stratification. **Results:** Using consensus clustering of 31 core CCGs in TCGA-LIHC and three independent validation cohorts (CHCC, LIRI, LICA), we identified three reproducible subtypes—Cluster-1 (metabolic–quiescent), Cluster-2 (transition–intermediate), and Cluster-3 (proliferation–inflammatory)—which were recapitulated across cohorts and showed distinct overall survival (Cluster-3 worst; log-rank *p* values significant across datasets). Multi-omic characterization revealed that Cluster-3 exhibits the highest tumor mutational burden and CNV burden with enrichment of TP53/AXIN1/TERT alterations, strong activation of cell-cycle, E2F, and G2M programs, and an immune-hot yet immunosuppressed microenvironment enriched for TAMs, Tregs and MDSCs. By contrast, Cluster-1 shows relative genomic stability, dominant hepatic metabolic signatures (fatty-acid oxidation, bile-acid and xenobiotic metabolism) and an immune-cold phenotype. Single-cell mapping linked ALAS1 expression to malignant hepatocytes predominating in Cluster-1, whereas NONO and CSNK1D localized to stromal (CAFs/TECs) and both malignant/immune compartments respectively in Cluster-3, providing a cellular mechanism for subtype-specific metabolism, angiogenesis and immune modulation. Finally, a nine-gene CCG-based RiskScore validated prognostic stratification and drug-sensitivity predictions indicated subtype-specific therapeutic vulnerabilities (notably increased predicted TKI sensitivity in Cluster-3). **Conclusion:** In conclusion, this study proposes a robust circadian rhythm-based molecular classification of hepatocellular carcinoma, revealing three biologically and clinically distinct subtypes characterized by divergent genomic alterations, metabolic programs, immune microenvironment states, and prognostic patterns. By integrating bulk and single-cell transcriptomic data, we identify subtype-specific roles of key circadian regulators—including ALAS1, NONO, and CSNK1D—in shaping tumor metabolism, proliferation, stromal remodeling, and immune suppression. These findings highlight circadian dysregulation as a potential upstream factor associated with HCC heterogeneity and provide a conceptual framework for developing subtype-tailored mechanistic studies and circadian-informed therapeutic strategies.

## 1. Introduction

Hepatocellular carcinoma (HCC), one of the most frequently diagnosed solid malignancies worldwide, continues to pose a profound global health challenge with its steadily rising incidence. According to the latest GLOBOCAN 2022 estimates, liver cancer is the sixth most commonly diagnosed cancer and the third leading cause of cancer death globally, with approximately 866,000 new cases and 759,000 deaths annually [1]. The primary risk factors contributing to HCC pathogenesis include chronic infections with hepatitis B virus (HBV) or hepatitis C virus (HCV), heavy alcohol consumption, and increasingly, non-alcoholic fatty liver disease (NAFLD)—recently redefined as metabolic-associated steatotic liver disease (MASLD)—which is associated with metabolic syndrome and obesity [2]. While the exact pathogenesis of HCC remains enigmatic despite its escalating incidence and mortality rates, emerging evidence underscores the integral role of CCGs in its development. Circadian clock genes (CCGs) are fundamental regulators of hepatic metabolic and inflammatory processes. Emerging research indicates that the core circadian machinery—including BMAL1, CLOCK, and PER—directly orchestrates hepatocyte proliferation, DNA repair, and apoptosis. Dysregulation of these CCGs creates a pro-tumorigenic environment that facilitates the malignant transformation of hepatocytes and promotes tumor progression via the rewiring of metabolic pathways [3,4,5,6,7].

The circadian rhythm, a 24 h internal biological cycle, governs multiple physiological processes, encompassing sleep, metabolism, and immune function. Essential constituents of the molecular clock mechanism, biological clock genes such as Clock, Bmal1, Per, and Cry, actively regulate the generation of these biological rhythms [7,8,9]. Aberrant expression or mutations of circadian clock genes were associated with a variety of diseases, including cancer [10,11].

Previous studies have shown that the expression levels of circadian clock genes are dysregulated in HCC tissues compared to normal liver tissues. In HCC tissues, downregulation of Clock and Bmal1 expression was observed, while upregulation of Per1, Per2, and Cry1 expression was detected. Additionally, mutations in Clock, Bmal1, and Per2 were found in patients with HCC, and these mutations were associated with poor prognosis [12].

While existing HCC classifications focus on terminal phenotypic or immune states, they often overlook the upstream regulatory clocks that synchronize metabolism and the cell cycle. Our circadian-based subtyping identifies the fundamental temporal dysregulation that drives these downstream heterogeneities, providing a more mechanistic framework for therapeutic stratification.

In HCC, alterations in circadian gene expression and mutations in clock components have been observed; however, whether circadian dysregulation can serve as a new axis of tumor stratification remains unclear. To address this, we systematically analyzed CCG expression across four independent HCC cohorts, identified robust CCG-based molecular subtypes, and performed multi-omics characterization to uncover their genomic, functional, immunological, and therapeutic distinctions.

## 2. Materials and Methods

### 2.1. Study Cohorts from Public Records

An extensive examination of four hepatocellular carcinoma cohorts was undertaken to pinpoint potential biomarkers. Integration of four RNA sequencing datasets (TCGA-LIHC, CHCC, LIRI, and LICA) facilitated a detailed analysis of HCC’s molecular characteristics. Rigorous data preprocessing protocols were implemented to maintain data quality and uniformity. Pre-normalized data for TCGA-LIHC were acquired from UCSC Xena, with clinical information for the same cohort sourced from the supplementary files of relevant publications. Further enriching the study, SNV data and copy number variation (CNV) profiles were obtained from the TCGA database. To address batch effects and facilitate data integration, non-tumor samples were excluded, and the ComBat method was applied for data harmonization [13].

### 2.2. TCGA-LIHC Clustering Analysis

The TCGA-LIHC dataset was designated as the training set for the purpose of streamlining subsequent analyses, with the remaining three datasets utilized as external validation sets. Consensus clustering analysis, incorporating mutations, CNV, and gene expression profiles from the training set along with a selection of genes, was conducted to stratify the data [14]. To avoid ambiguity, we clarify that genomic alterations (mutations and CNV) were analyzed post-clustering to delineate subtype-specific biological properties. To ascertain the optimal number of clusters (k), 500 iterations were performed, examining clustering possibilities ranging from 2 to 5. The average silhouette width (ASW) served as the metric for assessing the quality of the clustering for each k value.

### 2.3. Establishment of RiskScore via LASSO Cox Regression

Genes selected based on univariate Cox analysis were further incorporated into LASSO Cox regression, with coefficients calculated based on the optimal lambda value [15]. A scoring system was established on the basis of gene coefficients, and we referred to its result as “RiskScore”. Patients were categorized into high-risk and low-risk groups based on the median value of RiskScore. The calculation formula for RiskScore is as follows:

riskScore = 0.20212713 × CSE1L + 0.16090797 × NONO + 0.05876967 × PPARG + (−0.18357626) × ALAS1 + 0.08584138 × PRKAA2 + 0.07390319 × ARNTL2 + 0.10771332 × CSNK1D + (−0.24376618) × CRY2 + 0.05699038 × CSNK2A2

### 2.4. Single-Cell RNA-Seq Analysis

The single-cell RNA-Seq data of GSE15130 was downloaded and processed utilizing the “Seurat” package in R (4.5.1 version), focusing on samples with IDs S001-S005 [16]. The data was normalized using the “NormalizeData” and “FindVariableGenes” functions, identifying 2000 highly variable genes in the process. Principal component analysis was conducted using the “FindNeighbors” and “FindClusters” functions. The expression profiles of genes constituting the RiskScore were depicted using violin plots through the “vlnplot” function.

### 2.5. Pathway and Gene Enrichment Analysis

The clusterProfiler package (4.18.4 version), in conjunction with the Gene Ontology resource, was utilized to conduct Gene Set Enrichment Analysis (GSEA), identifying pathway differences across Biological Processes (BPs), Cellular Components (CCs), and Molecular Functions (MFs) [17]. The emapplot function was then applied to the GSEA results to produce enrichment plots, which facilitated the detection of gene set clusters with shared functional modules. A manual examination of the enrichment outcomes enabled the extraction of primary biological themes for each cluster, which were subsequently annotated on the plots.

For the assessment of pathway or label enrichment among the three subtypes, Gene Set Variation Analysis (GSVA) was performed using the GSVA package (2.4.7 version) [18]. The gene sets used in the analysis, including Hallmark and other pathway gene sets, were obtained from the Molecular Signatures Database (MSigDB). Enrichment scores for each gene set were calculated and differential analysis was conducted to determine variations between the three subgroups.

### 2.6. Statistical Analysis

All statistical analyses were conducted using the R language (version 4.3.1). The Wilcoxon rank-sum test was utilized for comparisons between two groups, while one-way analysis of variance facilitated comparisons among multiple groups. Pearson correlation analysis was applied to compute correlation coefficients. Notations for statistical significance were as follows: NS indicates not statistically significant; * *p* < 0.05; ** *p* < 0.01; *** *p* < 0.001, with a *p*-value < 0.05 deemed to indicate statistical significance. To control for the False Discovery Rate (FDR) associated with multiple hypothesis testing in high-throughput transcriptomic data, *p*-values for differential expression analysis (DEA) and Gene Set Enrichment Analysis (GSEA) were adjusted using the Benjamini–Hochberg (BH) method. An adjusted *p*-value (FDR) < 0.05 was strictly applied as the threshold for identifying significantly differentially expressed genes and enriched pathways, thereby ensuring the robustness of our inferential predictions and minimizing Type I errors.

## 3. Results

### 3.1. Circadian Core Clock Gene Analysis Reveals Prognostic Clusters and Biological Significance in Hepatocellular Carcinoma

**Figure 1:**
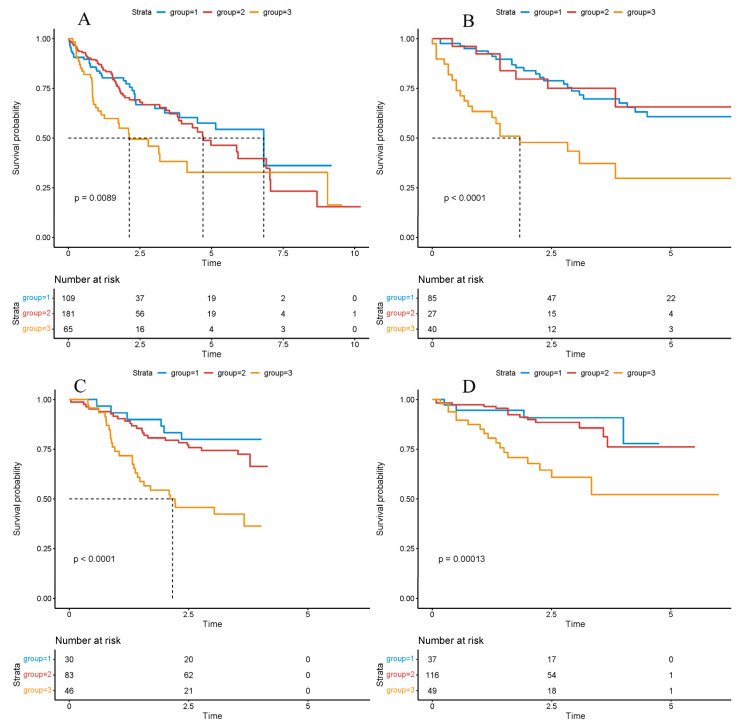
Survival analysis based on the cluster model. Survival analysis based on the cluster model in the TCGA ((**A**), *p*-value = 0.0089), LICA ((**B**), *p*-value < 0.0001), CHCC ((**C**), *p*-value < 0.0001), LIRI ((**D**), *p*-value = 0.0013) datasets. Significance was determined using the log-rank test. Hazard ratios (HRs) and 95% confidence intervals (CIs) were calculated to evaluate the effect size. *p* < 0.05 was considered statistically significant.

We acquired transcriptomic and clinical data from The Cancer Genome Atlas—Liver Hepatocellular Carcinoma (TCGA-LIHC) cohort for primary analysis. Following standard survival analysis quality control, samples with follow-up shorter than 30 days were excluded, leaving 355 evaluable HCC cases for downstream analyses. Based on prior literature curation of canonical clock components, we compiled a list of 31 core circadian clock genes (CCGs) for downstream clustering and association analyses. Unsupervised consensus clustering of the 31 CCG expression profiles was performed to identify reproducible molecular subtypes based on circadian program activity. The CancerSubtypes/ConsensusClustering framework was used to ensure clustering stability. Consensus clustering in TCGA-LIHC identified three discrete subgroups (hereafter Cluster-1, Cluster-2, and Cluster-3); the same clustering approach was applied to three independent HCC cohorts (LIRI, LICA, and CHCC) to validate reproducibility. The optimal cluster number (k = 3) was selected based on inspection of the cumulative distribution function (CDF) and consensus heatmaps, which indicated maximal cluster stability and separation (Figure 1A–D). The complete list of the 31 core circadian clock genes (CCGs) could be found at the Supplementary Materials Table S2.

**Figure 2:**
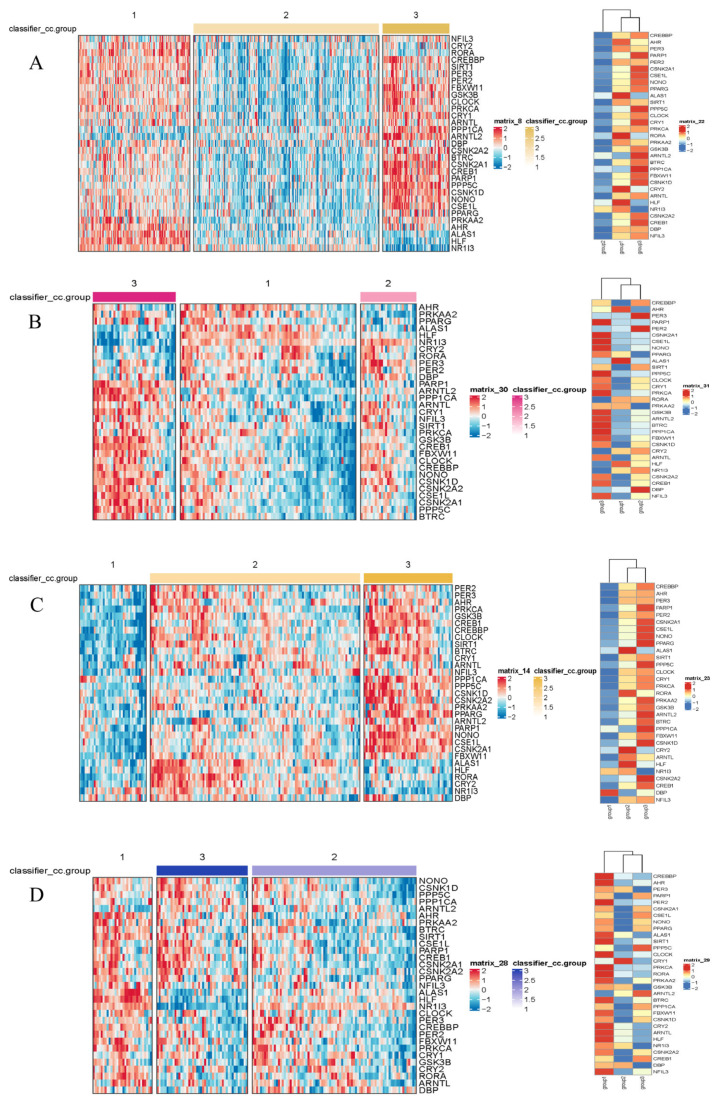
Heatmaps of gene expression by subtype in the TCGA (**A**), LICA (**B**), CHCC (**C**), and LIRI (**D**) datasets. The color scale represents the Z-score-normalized gene expression levels. Differential expression analysis among the identified subtypes was performed using the limma package (3.66.0 version). Genes displayed in the heatmaps met the criteria of an adjusted *p*-value (Benjamini–Hochberg method) < 0.05, ensuring statistical significance across all four independent cohorts.

Overall survival across the three clusters was next compared using Kaplan–Meier estimation and log-rank testing to assess prognostic stratification. Across TCGA-LIHC and the three external cohorts (LIRI, LICA, CHCC), patients assigned to Cluster-3 consistently exhibited the poorest overall survival, whereas Cluster-1 and Cluster-2 showed comparably improved outcomes (Figure 2A–D), underscoring cross-cohort robustness.

**Figure 3:**
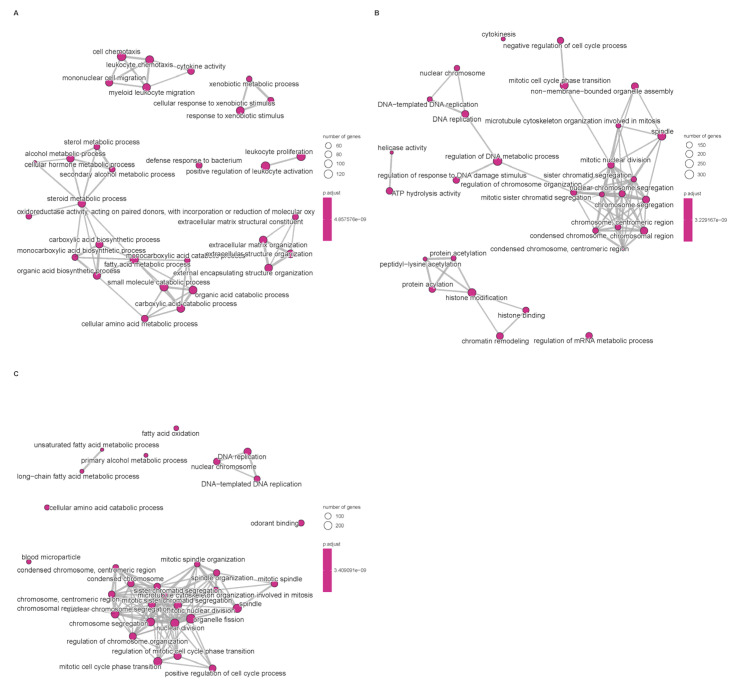
Enrichment of signaling pathways based on TCGA database. (**A**): Disorders of bile acid synthesis and biliary transport/Fatty acid omega-oxidation. (**B**): Cholesterol metabolism. (**C**): Cell cycle/Retinoblastoma gene in cancer. Pathway enrichment significance was determined based on Normalized Enrichment Scores (NES) and adjusted *p*-values (FDR). Only terms with |NES| > 1 and FDR < 0.05 are visualized.

Differential expression analysis between subtypes was performed using the limma pipeline and resulting gene sets were subjected to Gene Ontology (GO) and KEGG pathway enrichment to reveal biological themes associated with each cluster (Figure 3). GO enrichment revealed that subtype-associated DEGs are significantly over-represented in processes such as nuclear division, mitotic cell cycle transitions, and xenobiotic stimulus response, implicating coordinated regulation of proliferation and hepatic metabolic/detoxification functions. These enrichments suggest that altered proliferative programs and impaired xenobiotic metabolism contribute to HCC pathogenesis; in particular, up-regulation of mitosis and cell cycle modules likely underpins the aggressive phenotype observed in the poor-prognosis cluster. Conversely, enrichment of xenobiotic-metabolism and detoxification pathways may reflect altered tumor–host interactions affecting antigen processing or immune recognition, thereby contributing to immune evasion in certain subtypes.

Having confirmed that the three circadian rhythm-related subtypes display markedly different prognoses across multiple independent HCC cohorts, we next aimed to systematically dissect the biological basis underlying their heterogeneity. Specifically, the subsequent analyses focus on four dimensions—genomic alterations, functional pathway activity, immune microenvironment composition, and treatment sensitivity—to comprehensively characterize the unique molecular and cellular features of Cluster-1 (metabolic–quiescent type), Cluster-2 (transition–intermediate type), and Cluster-3 (proliferation–inflammatory type).

### 3.2. Prognostic Significance of Core Clock Genes in Liver Cancer: Insights from Cox Regression and LASSO Analyses

**Figure 4:**
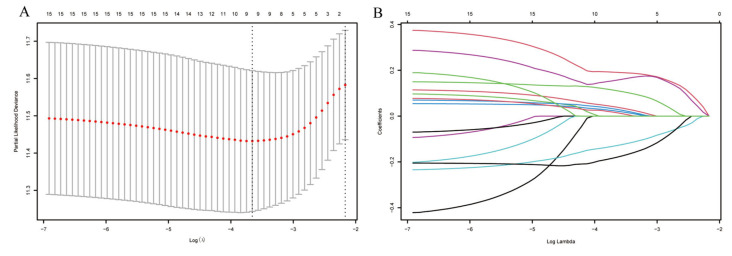
Analysis using the LASSO regression model. (**A**) Selection of the tuning parameter (λ) through cross-validation in the LASSO regression model. (**B**) LASSO coefficient profile plot.

**Figure 5:**
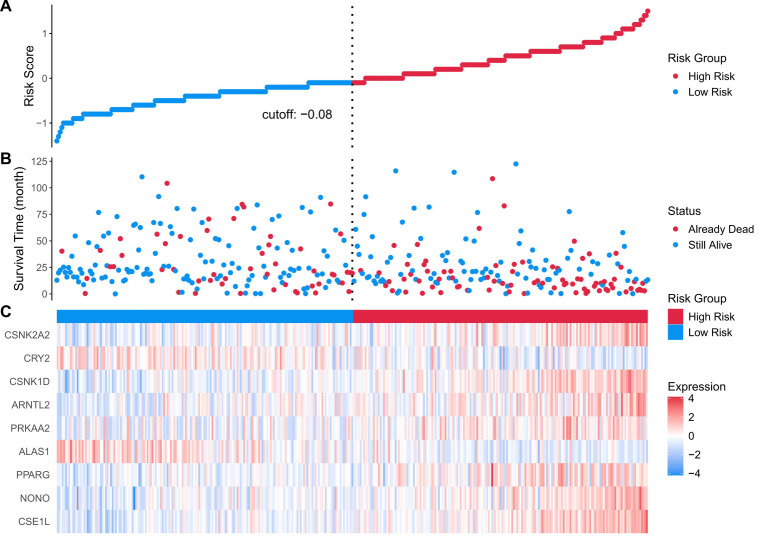
(**A**) risk score distribution heatmap for the TCGA cohort; (**B**) patient status heatmap for the TCGA cohort; (**C**) nine prognostic genes’ expression heatmap for the TCGA cohort. Statistical significance was determined using the log-rank test. Hazard ratios (HRs) and 95% confidence intervals (CIs) were calculated to evaluate the effect size. *p* < 0.05 was considered statistically significant.

**Figure 6:**
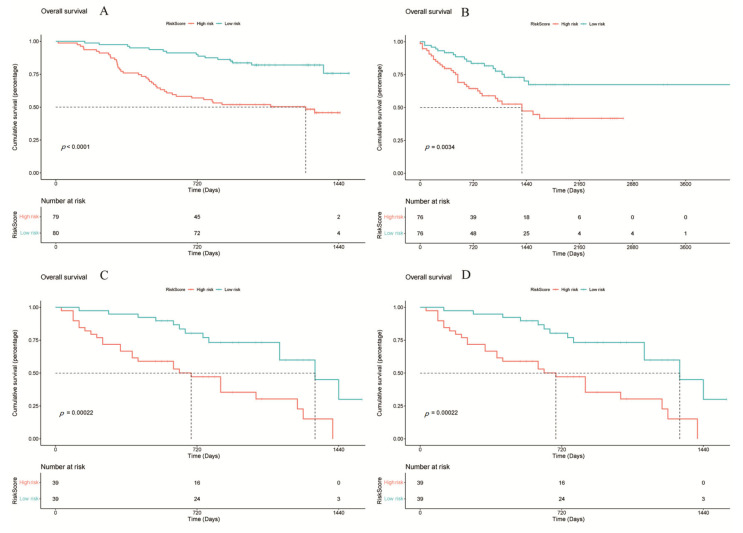
Survival analysis of different cohorts based on the RiskScore model. (**A**) TCGA, (**B**) LICA, (**C**) CHCC, (**D**) LIRI. Statistical significance was determined using the log-rank test. Hazard ratios (HRs) and 95% confidence intervals (CIs) were calculated to evaluate the effect size. *p* < 0.05 was considered statistically significant.

Univariate Cox proportional hazards analyses identified 15 of the 31 CCGs as significantly associated with overall survival (*p* < 0.05), indicating broad prognostic relevance of clock components. To derive a parsimonious prognostic signature, LASSO-penalized Cox regression was applied, yielding a nine-gene circadian signature associated with survival (Figure 4A,B). Model coefficients indicated that higher expression of CSE1L, NONO, PPARG, PRKAA2, ARNTL2, and CSNK1D was associated with increased risk, whereas elevated ALAS1 and CRY2 expression exhibited protective associations. Specifically, CSE1L and NONO are known to facilitate nucleocytoplasmic transport and RNA splicing of cell cycle regulators, thereby driving rapid tumor proliferation. PPARG and PRKAA2 are implicated in metabolic reprogramming that supports the energy demands of malignant cells. Conversely, ALAS1 is a rate-limiting enzyme in heme biosynthesis, and its high expression is a hallmark of well-differentiated, functional hepatocytes, suggesting it serves as a marker of lower malignancy. CRY2, a core negative feedback regulator of the clock, has been shown to promote the degradation of oncogenic proteins like c-Myc, thus explaining its protective role in HCC prognosis. Notably, CSE1L and NONO have been reported to promote tumor progression in other cancers [19,20,21,22,23], and CSNK1D has been implicated in HCC progression via Wnt/β-catenin signaling [4]. A composite RiskScore was computed from the nine-gene signature coefficients; patients were dichotomized at the median RiskScore into high- and low-risk groups for survival comparisons (Figure 5). In TCGA-LIHC the high-RiskScore group showed markedly reduced overall survival versus the low-RiskScore group; this prognostic separation was recapitulated in the three external validation cohorts (Figure 6A–D), supporting the signature’s robustness. We performed univariate and multivariate Cox regression analyses incorporating the RiskScore and clinical variables. The results, presented in Supplementary Table S1 and Figure S1, confirm that the RiskScore is a robust independent prognostic factor (HR > 1, *p* < 0.001). We generated ROC curves to assess the model’s predictive accuracy. These curves and their corresponding AUC values are provided in Supplementary Figure S2.

### 3.3. Genomic Alterations Distinguish the Three Circadian Subtypes

To clarify whether circadian-defined clusters harbor distinct genomic architectures, we integrated single-nucleotide variant (SNV) and copy-number variation (CNV) profiles from TCGA-LIHC and compared mutational and chromosomal instability patterns across subtypes.

We calculated the total number of non-synonymous SNVs per sample. Cluster-3 exhibited the highest TMB, significantly exceeding the levels observed in Cluster-1 and Cluster-2 (*p* < 0.001). In contrast, Cluster-1 displayed the lowest TMB, consistent with a genomically stable phenotype. These results support that Cluster-3 is characterized by heightened genomic instability, whereas Cluster-1 exhibits a relatively quiescent mutation landscape.

We next compared the frequencies of classical HCC driver mutations using Fisher’s exact test. Cluster-3 showed significantly elevated mutation rates in TP53, AXIN1 and TERT promoter regions (all *p* < 0.05). Cluster-1, conversely, was enriched for CTNNB1 mutations and displayed reduced TP53 alteration frequency. Cluster-2 showed intermediate mutation frequencies, reinforcing its transitional molecular status. These findings indicate that driver-gene perturbations parallel the biological behaviors observed in each subtype.

To further explore the biological link between genomic alterations and circadian dysregulation, we correlated the expression of our risk-related CCGs with the mutation status of key HCC drivers. We found that the expression of high-risk components, notably *NONO* and *CSE1L*, was significantly elevated in patients harboring *TP53* mutations (*p* < 0.05). In contrast, the protective gene *ALAS1* exhibited a positive correlation with *CTNNB1* mutation status, a hallmark of the metabolically active HCC subtype. These findings suggest that specific oncogenic driver mutations may synergize with the disruption of core circadian genes to shape the distinct molecular landscapes and clinical trajectories of the identified subtypes.

Genome-wide CNV burden was quantified as the fraction of altered genomic segments. Cluster-3 again showed the highest CNV load, with pervasive chromosomal amplifications and deletions, whereas Cluster-1 maintained a stable CNV profile (*p* < 0.001). GISTIC2.0 analysis revealed subtype-specific CNV events, including recurrent gains on 1q and 8q and losses on 4q and 17p in Cluster-3. Cluster-1 exhibited minimal focal alterations, consistent with its “metabolic–quiescent” character.

To evaluate whether CNVs directly influence circadian clock gene dysregulation, we assessed CNV status for the nine core genes contributing to the RiskScore. CSE1L, NONO, and CSNK1D displayed significant copy-number amplification in Cluster-3, accompanied by elevated mRNA expression (Pearson R > 0.45, *p* < 0.01). In contrast, ALAS1 and CRY2 showed copy-number loss and reduced expression preferentially in Cluster-3. This phenomenon suggests that in the highly unstable genomic landscape of Cluster-3 (characterized by high CNV burden), these protective circadian genes are located within common deletion regions. The physical loss of these genomic segments (CNV loss) provides a primary mechanism for the transcriptional silencing of ALAS1 and CRY2. The deficiency of these “circadian brakes” likely contributes to the loss of hepatocyte identity and the acquisition of a hyper-proliferative phenotype in Cluster-3 patients. These patterns illustrate that CNV-driven transcriptional dysregulation may contribute to subtype-specific circadian phenotypes and their downstream functional consequences. Together, these multi-layered genomic analyses establish that Cluster-3 is a genomically unstable, mutation-rich, and CNV-driven subtype, whereas Cluster-1 represents a metabolically oriented but genetically stable tumor state, with Cluster-2 occupying an intermediate space between them.

### 3.4. Single-Cell RNA Sequencing Reveals Differential Expression of Core Clock Genes Across Distinct Hepatocellular Carcinoma Cell Types

**Figure 7:**
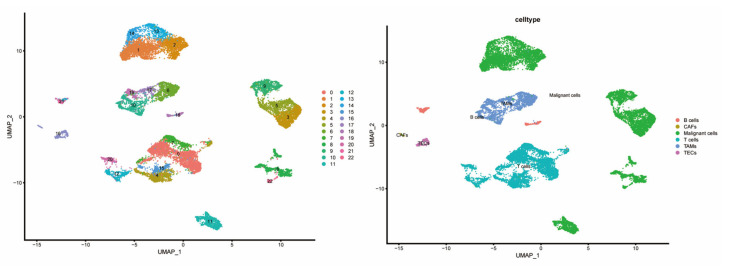
Composition and distribution of single cells from GSE151530.

To explore the cell-type-specific expression of CCGs, we analyzed single-cell RNA-seq data from HCC specimens (GSE151530), applying established single-cell workflows for dimensionality reduction and clustering (Figure 7). We used the publicly available GSE151530 dataset, which contains paired tumor and adjacent normal liver single-cell profiles; five tumor samples were selected for detailed analysis. Data preprocessing, normalization, variable gene selection, PCA, clustering and UMAP visualization were implemented using Seurat’s standard pipeline. Clustering resolved 23 transcriptionally distinct cell clusters, which were annotated into six major cell types—B cells, cancer-associated fibroblasts (CAFs), malignant epithelial cells, T cells, tumor-associated macrophages (TAMs), and tumor endothelial cells (TECs)—based on canonical marker expression.

**Figure 8:**
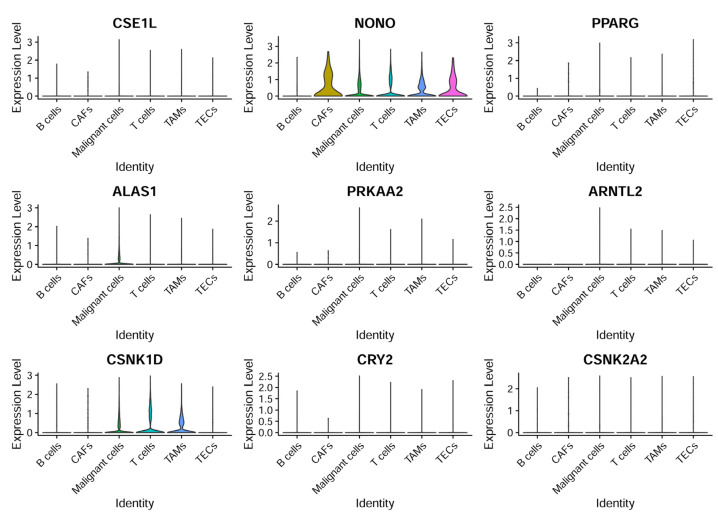
CCG expression based on single-cell sequencing analysis. The expression patterns of nine specific genes across different cell types.

CCG expressions were then profiled across the annotated cell types to identify cell-type-restricted and ubiquitous clock components (Figure 8). It was observed that NONO was broadly expressed across cell compartments with enrichment in CAFs and TECs, ALAS1 expression was largely confined to malignant hepatocytes, and CSNK1D was elevated in malignant cells as well as in T cells and TAMs—suggesting compartmentalized roles of specific CCGs in tumor and stromal compartments.

### 3.5. Integration of Single-Cell Mapping and Bulk Subtyping Reveals Cell-State Determinants and Intercellular Communication Mechanisms

To elucidate the cellular foundations of the three circadian-defined subtypes, we integrated single-cell RNA-seq data (GSE151530) with bulk transcriptomic subtyping and systematically mapped cell-type-specific expression of key CCGs.

Single-cell expression mapping revealed that ALAS1 demonstrates highly restricted expression in malignant hepatocytes, consistent with a metabolic-dominant tumor state. Moreover, the scRNA-seq composition showed markedly reduced proportions of T cells, macrophages, and endothelial cells within the ALAS1-high population. This cellular composition corroborates the “immune-cold” microenvironment observed in bulk data and suggests that Cluster-1 is shaped by intrinsically metabolically active but immunologically silent malignant cells.

Two CCGs—NONO and CSNK1D—formed important mechanistic links for Cluster-3:NONO was found enriched in CAFs and tumor endothelial cells (TECs), indicating that stromal components contribute to its inflammatory and angiogenic signatures; CSNK1D was highly expressed both in malignant epithelial cells and immune populations (T cells, TAMs). This dual-population expression pattern suggests that CSNK1D is significantly associated with both tumor proliferation (via Wnt/β-catenin signaling) and immune cell activation or suppression, potentially serving as a bridge that integrates “proliferation” and “inflammation,” the two dominant phenotypes of Cluster-3. Single-cell abundance analysis further confirmed an accumulation of TAMs and Tregs in Cluster-3, forming the cellular basis of the “immune-hot but immunosuppressed” ecosystem identified in bulk profiles.

Cluster-2 contained mixed contributions from both ALAS1-high malignant cells and NONO/CSNK1D-expressing stromal and immune populations. The relatively balanced distribution of metabolic, proliferative, stromal and immune cell states supports its role as a transitional intermediate subtype. This intermediate positioning is consistent across genomic, metabolic and immune layers, forming a coherent multiscale biological identity.

These integrated analyses construct a full evidence chain from bulk transcriptomics to cellular composition and molecular mechanisms, providing mechanistic explanations for the distinct tumor cell states and microenvironmental landscapes of the three circadian-defined subtypes.

### 3.6. Scissors Algorithm Analysis Reveals Distinct Immune Response Profiles and Functional Enrichment in the Training Set

**Figure 9:**
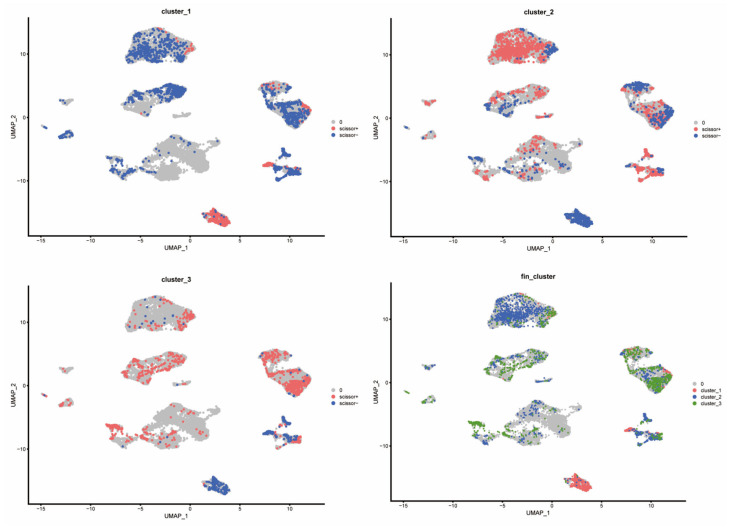
Distribution of different cluster cells in the scissors algorithm.

Applying the scissors algorithm to map bulk subtype labels from TCGA onto single-cell profiles revealed that the cells most strongly associated with the three circadian clusters were predominantly malignant epithelial cells (Figure 9). Mapping revealed clear differences in stromal and immune composition: Cluster-1 samples exhibited markedly reduced representation of T cells, TAMs, and TECs, whereas Cluster-3 samples were enriched for these cell classes—consistent with immune-cold vs. immune-active distinctions. Accordingly, we denote Cluster-1 as an immune-inactive (immune-cold) subtype, Cluster-3 as an immune-active (immune-hot) subtype, and Cluster-2 as an intermediate phenotype.

### 3.7. Immune Profiling and CCGs Analysis in HCC Clusters

**Figure 10:**
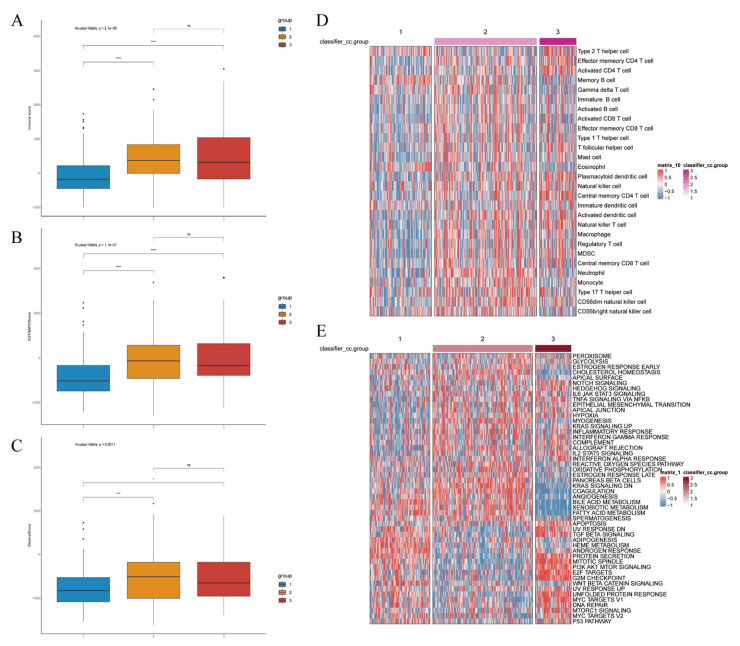
Clustering-based immune landscape and prediction of tumor response to immunotherapy. Immune score ((**A**), *p*-value < 0.001), ESTIMATE score ((**B**), *p*-value < 0.001), and stromal score ((**C**), *p*-value = 0.0001). (**D**) Inter-group expression patterns based on clustering of various cell types. (**E**) Based on clustering vrious functional pathways and their corresponding expression patterns. Differences between the three molecular subtypes were assessed using the Kruskal–Wallis test. For multiple comparisons, *p*-values were adjusted using the Benjamini–Hochberg (FDR) method. ns indicates not statistically significant; * *p* < 0.05; *** *p* < 0.001; **** *p* < 0.0001. Effect sizes for pathway enrichment are reported as Normalized Enrichment Scores (NES) in Supplementary Tables S3 and S4.

**Figure 11:**
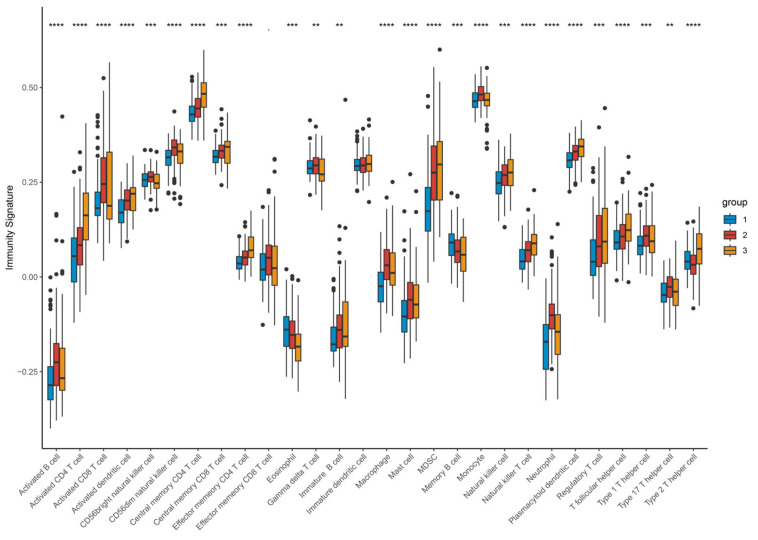
Expression profile of different types of infiltrating immunocytes based on clustering. Upregulated (red stripe) and downregulated (blue stripe) immune cells are presented according to immunocyte infiltration analysis. ** *p* < 0.01; *** *p* < 0.001; **** *p* < 0.0001. Differences between the three molecular subtypes were assessed using the Kruskal–Wallis test. For multiple comparisons, *p*-values were adjusted using the Benjamini–Hochberg (FDR) method. Effect sizes for pathway enrichment are reported as Normalized Enrichment Scores (NES) in Supplementary Tables S3 and S4.

We quantified the immune and pathway signatures at the sample level using single-sample GSEA (ssGSEA) to obtain immune infiltration and activation scores for each TCGA sample. The article further estimated immune-cell composition with CIBERSORT and compared scores across the three clusters (Figure 10A–E), enabling cell-type-level comparisons. Consistent with single-cell mapping, Cluster-1 displayed an overall immune-inactive profile, Cluster-3 an immune-activated profile, and Cluster-2 an intermediate state. CIBERSORT and ssGSEA demonstrated that Cluster-3 exhibits broad activation of T-cell and myeloid compartments, with significantly increased infiltration of activated CD4^+^ T cells, central memory T cells, follicular helper T cells, activated dendritic cells, NK-like T cells, and myeloid-derived suppressor cells (all *p* < 0.001). Although such extensive lymphocyte recruitment suggests an immune-hot phenotype, the concurrent enrichment of Tregs and MDSCs indicates a counterbalancing immunosuppressive program. Thus, Cluster-3 represents an immune-inflamed but immunosuppressed ecosystem, consistent with the phenotype observed in both bulk and single-cell analyses. (Figure 11).

**Figure 12:**
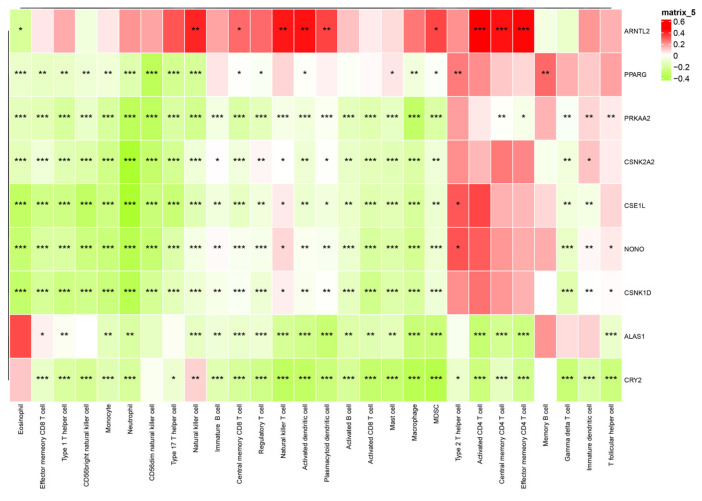
Interaction heatmap of gene expression across various immune cells. The association between CCG expression and immune cell infiltration was evaluated using Pearson correlation coefficients (R), which serve as the effect size. Statistical significance was assessed using correlation *p*-values adjusted for multiple testing. * *p* < 0.05; ** *p* < 0.01; *** *p* < 0.001.

Correlation analyses between CCG expression and immune-cell abundance indicated that ARNTL2 correlates positively with broad immune infiltration, whereas several other clock genes show inverse correlations with immune cell counts (Figure 12), implying gene-specific links to tumor immune architecture.

**Figure 13:**
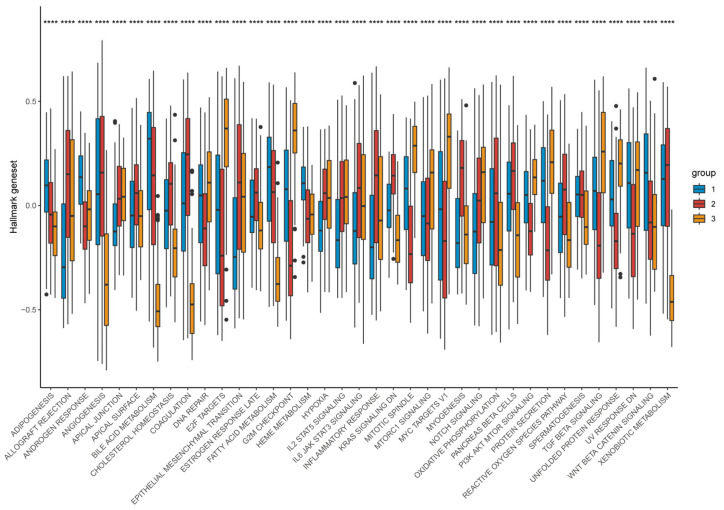
Distribution of hallmark geneset expression across different groups. Differences between the three molecular subtypes were assessed using the Kruskal–Wallis test. For multiple comparisons, *p*-values were adjusted using the Benjamini–Hochberg (FDR) method. **** *p* < 0.0001. Effect sizes for pathway enrichment are reported as Normalized Enrichment Scores (NES) in Supplementary Tables S3 and S4.

Functional enrichment demonstrated that Cluster-1 was enriched for metabolic pathways including fatty-acid β-oxidation, bile-acid metabolism, and CYP/xenobiotic detoxification, accompanied by Wnt/β-catenin activation, explaining its immune-cold, metabolically driven phenotype. By contrast, Cluster-3 showed strong enrichment for E2F targets, G2M checkpoint, Notch signaling, IL-2/STAT5 signaling, hypoxia, and glycolysis—reflecting a highly proliferative, metabolically reprogrammed, and inflammatory tumor state. Cluster-2 maintained intermediate activation across these programs, consistent with its transitional nature (Figure 13).

### 3.8. Pseudotemporal Trajectory Analysis Reveals Developmental Dynamics of Malignant Cells

**Figure 14:**
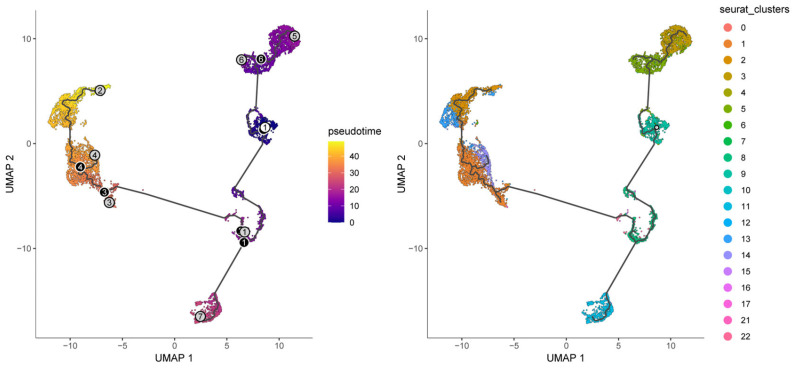
Pseudotime and developmental trajectory of the subpopulation of malignant cells.

**Figure 15:**
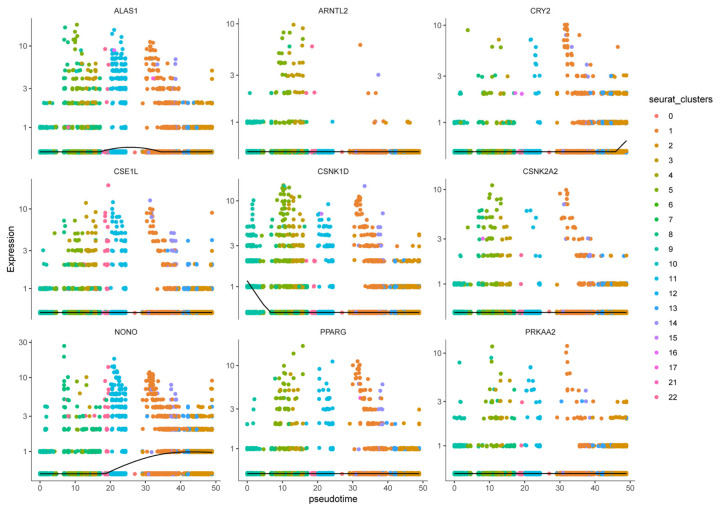
Expression patterns of nine prognostic genes in pseudo-timing of different cell clusters.

To interrogate malignant-cell developmental dynamics, Monocle3 pseudotemporal trajectory analysis was performed on malignant clusters to reconstruct putative differentiation paths [24]. Pseudotime mapping placed the 23 malignant clusters along ordered trajectories, enabling identification of branching events and putative progression directions (Figure 14). Trajectory inference highlighted key branch points and multiple developmental branches—potential nodes of phenotypic divergence that merit further functional validation. Cells grouped along the same branch displayed concordant transcriptional programs, supporting the biological coherence of inferred developmental states. Importantly, the nine prognosis-related genes exhibited dynamic expression changes along pseudotime, suggesting temporal regulation of prognostic programs during malignant progression. For example, CSNK1D expression progressively declined along the trajectory, ALAS1 peaked at intermediate pseudotime, and NONO and CRY2 increased in late-stage pseudotime, implying stage-specific roles of these CCGs during tumor cell evolution (Figure 15).

## 4. Discussion

In this study, we established and validated a robust three-class circadian rhythm-related molecular classification of hepatocellular carcinoma (HCC) across four independent transcriptomic cohorts. By integrating multi-omics alterations, functional pathway activities, tumor microenvironment characteristics, and single-cell transcriptomic evidence, we delineated a comprehensive and multilayered biological landscape driven by circadian clock gene (CCG) dysregulation. Our findings reveal a hierarchical evidence chain—from genomic alterations and transcriptional programs to cellular states, immune ecosystem remodeling, and ultimately clinical prognosis—indicating that circadian disruption constitutes a fundamental axis of HCC heterogeneity.

Several prior studies have proposed molecular subtypes or prognostic signatures for HCC based on circadian clock genes (CCGs). However, our study provides several incremental and transformative advances. Methodologically, while earlier models relied primarily on bulk transcriptomic data for risk stratification, we integrated single-cell RNA-sequencing (scRNA-seq) with multi-omic bulk data across four independent cohorts. This approach allowed us not only to identify subtypes but also to achieve cellular-level validation of our findings. Biologically, our study transcends simple prognostic prediction by delineating the niche-specific roles of key regulators. For instance, while previous models identified *NONO* as a generic risk factor, our single-cell mapping specifically linked its elevation to the stromal and immune compartments (CAFs/TECs) of the aggressive Cluster-3, while identifying malignant hepatocyte-restricted *ALAS1* expression as a hallmark of the metabolic Cluster-1. By defining these cell-type-specific circadian roles, our study provides a more granular mechanistic framework for understanding the “metabolic-to-inflammatory” transition in HCC, which was not fully explored in prior stratification efforts.

The three molecular subtypes exhibited strikingly distinct biological identities. Cluster-1 was characterized by a genomically stable profile, dominant fatty acid oxidation and bile acid metabolism signature, and minimal immune infiltration, forming a typical metabolic-driven, immune-cold phenotype. In contrast, Cluster-3 displayed the highest tumor mutational burden, enriched TP53/AXIN1/TERT alterations, extensive CNV amplifications and deletions, and markedly activated cell-cycle and DNA repair pathways. This subtype also exhibited dense infiltration of T cells, macrophages, and immunosuppressive regulatory populations, generating an immune-hot but immunosuppressed microenvironment. Cluster-2 occupied an intermediate position across almost all dimensions, representing a biological continuum between the metabolic and proliferative extremes. These patterns suggest that circadian dysregulation is closely linked to a coordinated rewiring of tumor metabolism, genomic instability, and immune dynamics.

The integration of bulk and single-cell transcriptomic data further elucidated the mechanistic underpinnings of each subtype. The hallmark gene ALAS1, specifically expressed in malignant hepatocytes at single-cell resolution, accounted for the metabolic dominance and immune-excluded state of Cluster-1. Conversely, NONO, enriched in cancer-associated fibroblasts and tumor endothelial cells, provided a stromal basis for the angiogenic and inflammatory phenotype of Cluster-3. CSNK1D, expressed across both malignant and immune cells, emerged as a pivotal molecular “bridge” connecting proliferative signaling (e.g., Wnt/β-catenin activation) with immune regulation. These cell-type-specific patterns were fully consistent with the functional and clinical features observed in bulk data, reinforcing the biological validity of the circadian-defined subtypes.

Another important mechanistic insight emerging from our analysis is the dual-compartment role of CSNK1D, which emerged as a candidate molecular bridge associated with both proliferative and inflammatory programs in Cluster-3. Notably, our findings highlight the critical interplay between NONO and the cell cycle. NONO (Non-POU domain-containing octamer-binding protein) has been reported to stabilize the mRNA of key G2/M checkpoint genes and E2F targets. In Cluster-3, the high expression of NONO likely acts as a molecular bridge, coupling circadian rhythm disruption with accelerated mitotic entry. This interaction suggests that NONO not only disrupts the internal clock but also directly fuels the proliferative engine of HCC, making it a potential therapeutic vulnerability in the most aggressive molecular subtype. Prior studies have shown that CSNK1D stabilizes Disheveled proteins to activate Wnt/β-catenin signaling and promote tumor progression [4]. Our single-cell data extend this concept by revealing that CSNK1D is not only highly expressed in malignant hepatocytes but also in T cells and TAMs, suggesting that it may coordinate tumor-intrinsic proliferation with microenvironmental immune modulation.

These findings raise the possibility that CSNK1D exerts its effects through multiple downstream axes. In malignant cells, CSNK1D may activate Wnt/β-catenin and cell cycle regulators, driving rapid proliferation. In immune cells, CSNK1D may influence cytokine signaling, T-cell activation states, or macrophage polarization, thereby shaping the inflammatory milieu. Such multi-lineage regulatory potential positions CSNK1D as a candidate master regulator of the dual “proliferation–inflammation” phenotype characteristic of Cluster-3 and highlights it as a promising target for subtype-specific therapeutic strategies.

The observed microenvironmental differences also have important therapeutic implications. The immunosuppressive yet inflamed phenotype of Cluster-3 suggests that immune checkpoint blockade alone may be insufficient but could be significantly enhanced by combining circadian-targeting agents, metabolic modulators, or anti-angiogenic therapy. In contrast, the immune-cold and metabolically dominant phenotype of Cluster-1 may benefit more from tyrosine kinase inhibitors or metabolic–circadian combination strategies. These insights support a paradigm in which chronotherapy, or treatment scheduling aligned with circadian biology, may require subtype-specific timing strategies to optimize therapeutic outcomes.

Overall, this study provides the most comprehensive evidence to date that circadian rhythm dysregulation represents a significant upstream factor linked to molecular, metabolic, genomic, and immunologic diversity in HCC. The three circadian subtypes not only deepen our understanding of HCC biology but also provide a new framework for designing subtype-specific mechanistic studies and personalized therapeutic interventions. However, we acknowledge that the current findings are primarily based on retrospective multi-omics data and single-cell resolution mapping, and potential residual batch effects across cohorts may exist despite harmonization. Furthermore, due to the lack of experimental validation, the mechanistic roles of the identified circadian genes remain as associations. Future prospective studies and functional ‘wet-lab’ experiments are required to confirm the therapeutic potential of these circadian-informed strategies. Additionally, while the GSE151530 dataset provided valuable single-cell insights, it is limited by a small sample size (n = 5). Future studies incorporating larger, independent single-cell cohorts are necessary to validate these patient-level subtype mappings and ensure broader generalizability. Despite the comprehensive molecular subtyping and prognostic modeling presented here, several limitations warrant consideration. In this study, we focused on the integrated expression profiles of 31 core CCGs to define robust HCC clusters and a clinical RiskScore. However, we did not perform a systematic topological ‘Hub Gene’ analysis—such as through Protein–Protein Interaction (PPI) network ranking—to identify the specific master regulators within the circadian machinery that exert the greatest hierarchical influence on HCC progression. While our RiskScore has identified several high-impact genes like *NONO* and *CSNK1D*, a more granular network-centric analysis would further refine our understanding of the circadian hierarchy. We consider this a valuable direction for our subsequent research, where we plan to utilize advanced network pharmacology and systems biology approaches to pinpoint these hub CCGs as precision therapeutic targets. We humbly acknowledge several limitations. Most notably, the findings presented here are derived from retrospective multi-omics data, and direct experimental validation in cell lines or clinical samples was not performed in this current work. While our cross-validation strategy ensures a high degree of biological plausibility, future prospective studies incorporating in vitro and in vivo experimental models will be essential to validate these bioinformatic predictions and to translate these circadian insights into clinical practice. We are sincerely committed to conducting these follow-up experiments in our subsequent research to further elucidate the functional roles of the key circadian regulators identified in this study.

## 5. Conclusions

This study systematically characterized the landscape of circadian clock gene expression in hepatocellular carcinoma and established a reproducible three-class circadian molecular classification across multiple independent cohorts. The identified subtypes exhibit profound differences in genomic alterations, metabolic activity, proliferative signaling, immune microenvironment architecture, and clinical prognosis, revealing a previously underappreciated circadian-driven axis of HCC heterogeneity. Through integrated analyses of bulk transcriptomic data, single-cell expression patterns, SNV/CNV alterations, functional pathways, and immune signatures, we elucidate the subtype-specific cellular origins and mechanistic implications of key circadian regulators such as ALAS1, NONO, and CSNK1D.

These findings provide a conceptual and methodological foundation for future research. First, the functional roles of individual CCGs should be dissected within their appropriate subtype context rather than treating HCC as a homogeneous disease. Second, circadian-based combination therapies—particularly metabolic–circadian, immune–circadian, and TKI–circadian regimens—may be optimized by leveraging the subtype-specific biology. Third, clinical chronotherapy strategies may benefit from individualized timing schedules aligned with each circadian-defined tumor subtype.

In conclusion, our work establishes a comprehensive framework for understanding the temporal biology of HCC and lays the groundwork for developing circadian-informed precision therapeutic strategies. Future studies incorporating in vitro and in vivo experimental models will be essential to validate these bioinformatic predictions and to translate these circadian insights into clinical practice.

## Data Availability

The main text and Supplementary Materials have included all the data generated in the present study. The sources of the TCGA statistics used in this study can be found in the original TCGA.

## Supplementary Materials

The following supporting information can be downloaded at: https://www.mdpi.com/article/10.3390/biomedicines14030645/s1, Figure S1: Univariate and multivariate Cox regression analyses incorporating the RiskScore and clinical variables; Figure S2: Time-dependent ROC Curves; Table S1: Results of univariate and multivariate Cox regression analyses incorporating the RiskScore and clinical variables; Table S2: 31 circadian genes used to derive the clusters; Table S3: Enrichment scores (NES) and false discovery rate (FDR) values of GO analyses; Table S4: Enrichment scores (NES) and false discovery rate (FDR) values of KEGG analyses.
