# Supplementary figures and images for: Proposed Role of Circadian Clock Genes in Pathogenesis of HCC: Molecular Subtyping and Characterization
Source: Biomedicines. 2026 Mar 12;14(3):645. doi: 10.3390/biomedicines14030645 (PMC13024568; doi:10.3390/biomedicines14030645)

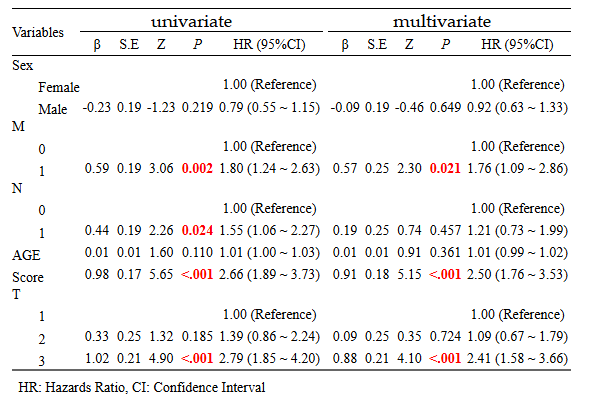

Supplement: Supplementary file 1 [file biomedicines-14-00645-s001.zip › Figure S1.png]

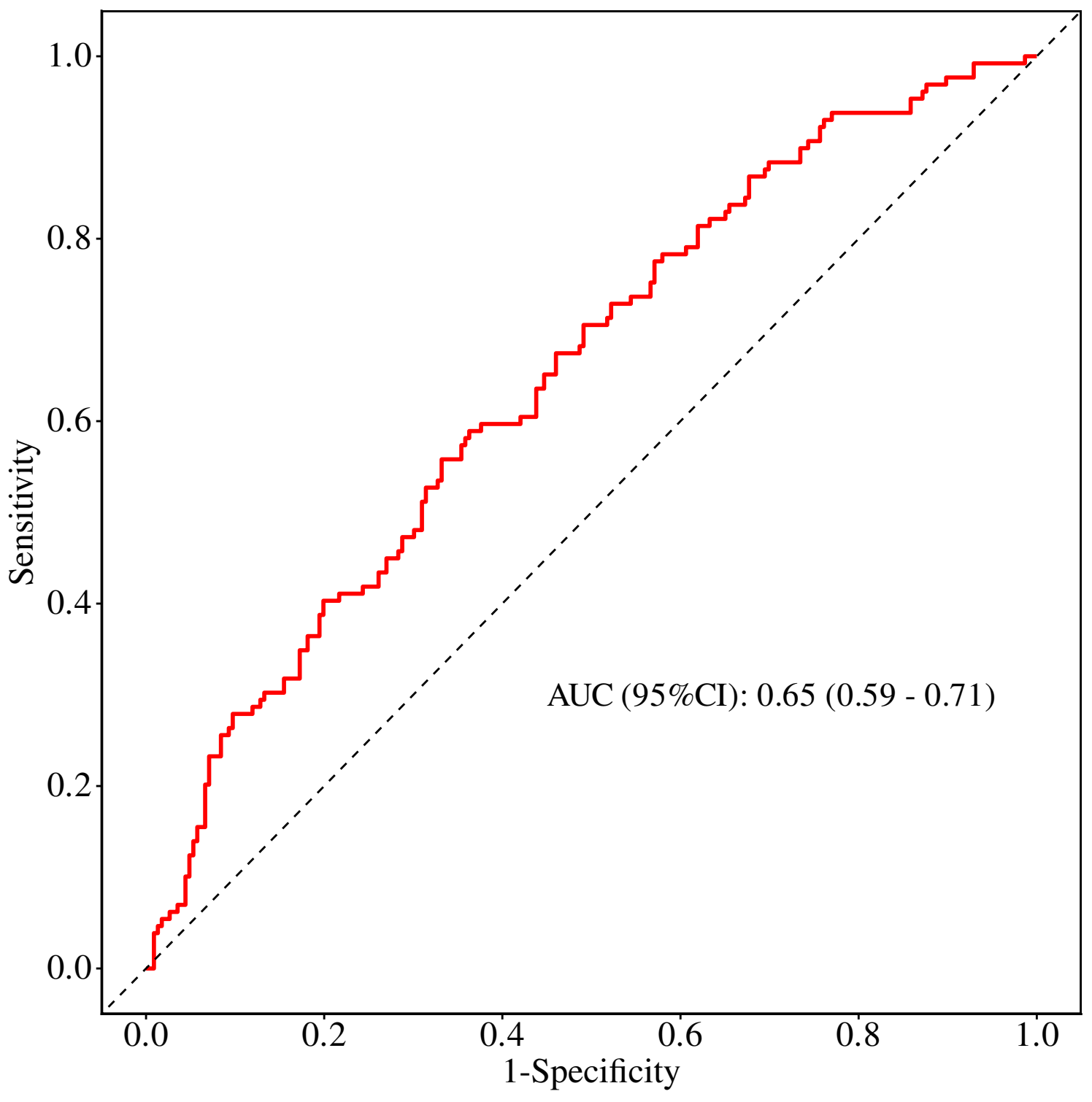

Supplement: Supplementary file 1 [file biomedicines-14-00645-s001.zip › Figure S2.pdf]
